# CMV Oophoritis in an AIDS Patient

**DOI:** 10.1155/S1064744995000627

**Published:** 1995

**Authors:** Michael S. Albert, Marvin S. Amstey

**Affiliations:** 1 Department of Obstetrics-Gynecology and Pathology The University of Rochester School of Medicine and Dentistry Rochester NY USA; 2 Department of Obstetrics and Gynecology Genessee Hospital 220 Alexander Street Suite 604 Rochester NY 14607 USA; 3 Department of Pathology The University of Rochester School of Medicine and Dentistry Rochester NY USA

## Abstract

*Background:* Disseminated cytomegalovirus (CMV) infection is relatively uncommon, occurring primarily in immunocompromised hosts and neonates. Patients with acquired immunodeficiency syndrome (AIDS) are the most common hosts, with symptoms secondary to lung and eye involvement. There have been no reports of symptomatic CMV infection of the pelvis in women.

*Case report:* This case is the first described of acute symptomatic CMV infection of the genital tract in a woman with AIDS. Her presenting symptoms were the result of acute CMV oophoritis. In addition, CMV was found in the endometrium and endosalpinx (an infected structure heretofore unreported).

*Conclusion:* The increasing prevalence and incidence of AIDS in women should make us aware of the possibility of opportunistic, symptomatic CMV pelvic infection.


Infectious Diseases in Obstetrics and Gynecology 3:202–204 (1995)
© 1996 Wiley-Liss, Inc.

### CMV Oophoritis in an AIDS Patient

**Michael S. Albert and Marvin S. Amstey**

*Departments of Obstetrics-Gynecology and Pathology (Ma.S.A.) and Pathology (Mi.S.A.), The University of Rochester School of Medicine and Dentistry, Rochester, NY*

Abstract

***Background:*** **Disseminated cytomegalovirus (CMV) infection is relatively uncommon, occurring primarily in immunocompromised hosts and neonates. Patients with acquired immunodeficiency syndrome (AIDS) are the most common hosts, with symptoms secondary to lung and eye involvement. There have been no reports of symptomatic CMV infection of the pelvis in women.**

***Case report:*** **This case is the first described of acute symptomatic CMV infection of the genital tract in a woman with AIDS. Her presenting symptoms were the result of acute CMV oophoritis. In addition, CMV was found in the endometrium and endosalpinx (an infected structure heretofore unreported).**

***Conclusion:*** **The increasing prevalence and incidence of AIDS in women should make us aware of the possibility of opportunistic, symptomatic CMV pelvic infection.** © 1996 Wiley-Liss, Inc.

Key words

Virus, pain, infection

Most healthy people beyond the neonatal period who become infected with cytomegalovirus (CMV) are asymptomatic. If symptoms do occur, they are usually mild and self-limiting. By age 35–40 years, approximately 50% of the population are seropositive for CMV.[1]

CMV enters the latent phase after a primary infection, with its DNA incorporated into the host's genome. Once infected, individuals probably carry the virus for life. Immunocompromised states, such as infection with the human immunodeficiency virus (HIV), can result in reactivation of CMV. Primary CMV infections in immunocompromised hosts do occur and are often severe; the reactivation of CMV also leads to pneumonia, colitis, chorioretinitis, encephalitis, and other manifestations.[2] CMV infection can lower T-cell ratios, suppress natural killer cell activity, and reduce T-cell proliferative response.[3] Up to 90% of acquired immunodeficiency syndrome (AIDS) patients develop active CMV infection during the course of their illness.

CMV oophoritis has been reported in 8 women immunocompromised from acute leukemia.[4] In addition, an autopsy series identified 4 women with CMV oophoritis with either a lymphoproliferative disorder or acute leukemia.[5] To our knowledge, CMV infection of the ovary has not been described either histologically or as a cause of morbidity in any woman with AIDS.

### CASE REPORT

P.H. was a 39-year-old white female who acquired HIV through heterosexual contact. She was diagnosed initially after developing pneumocystis pneumonia. Subsequently, she was treated with zidovudine and acyclovir. The patient's first admission was for a presumed pancreatitis secondary to zidovudine therapy. She was readmitted 8 months later for pneumococcal pneumonia. Shortly after, she developed severe, persistent metrorrhagia and anemia that was unresponsive to both medroxyprogesterone and danazol. An endometrial sampling demonstrated only a secretory endometrium. All

Address correspondence/reprint requests to Dr. Marvin S. Amstey, Department of Obstetrics and Gynecology, Genessee Hospital, 220 Alexander Street, Suite 604, Rochester, NY 14607.

Received July 18, 1995

Accepted October 20, 1995

Gynecological Case Report measures of clotting function were normal, and a vaginal hysterectomy was performed. The microscopic pathology of the uterine specimen demonstrated CMV endometritis as well as CMV inclusions in numerous small fibromyomata. The histology of the uterus identified CMV for the first time in this patient. Subsequently, she had CMV identified in her blood, sputum, and urine.

Ten days after her discharge, she was readmitted with severe pelvic and rectal pain. Examinations, barium enema, colonoscopy, and pelvic ultrasounds were not helpful in diagnosing her pain origin. She required high-dose narcotic analgesia. In less than a month, she was admitted to another hospital with an acute abdomen necessitating an ileocecal resection. A pathologic review of this specimen showed ischemic necrosis of the cecum with perforation. CMV was not identified in this specimen.

Her persistent pelvic pain prompted another admission in the same month. A colonoscopy with biopsies of the anastomotic site and the colon generally revealed CMV infection. The patient's pelvic and rectal pain continued, requiring parenteral morphine as well as repeated caudal blocks. She died 2 months later.

### Autopsy Findings

The important gross findings included diffuse consolidation of both lungs. The gastrointestinal tract was unremarkable. No ulcerations were identified.The ovaries were adherent to the pelvic sidewalls.

Microscopically, both lungs showed extensive acute and chronic CMV pneumonitis. CMV colitis and proctitis were also identified, but no ulcerations were found. The endometrium from the hysterectomy done three months earlier demonstrated CMV inclusions in the columnar epithelium (Fig. 1.). Both ovaries showed extensive involvement with CMV (Fig. 2).

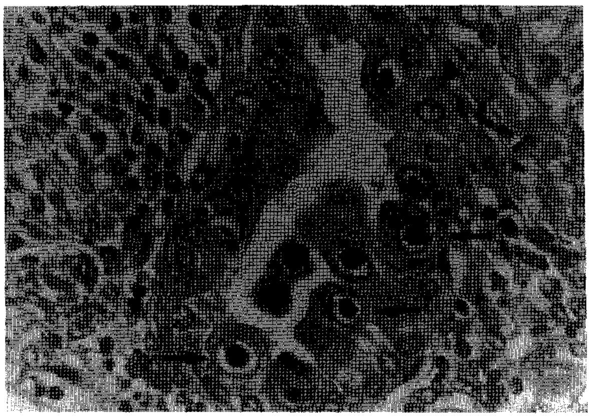

Fig. 1. Photomicrograph of CMV inclusions (arrows) in the columnar cells of the endometrium (× 120).

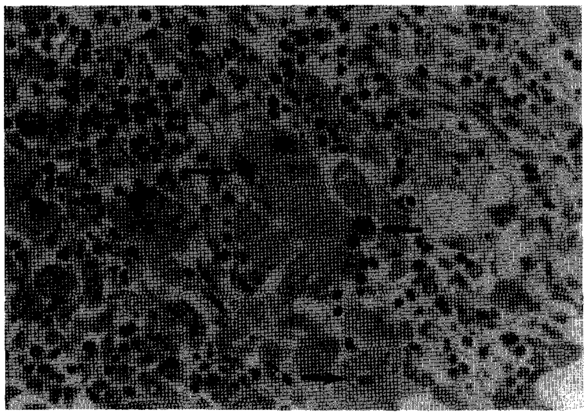

Fig. 2. Photomicrograph of CMV inclusions (arrows) in the stroma of the ovary (× 120).

### DISCUSSION

In immunocompromised AIDS patients, CMV is known to involve the lungs, adrenals, retina, brain, liver, esophagus, and colon. CMV pneumonitis alone has a mortality rate of nearly 90%.[6] An immune complex nephropathy has also been reported in association with CMV.[7] While CMV is known to infect the ovaries in patients immunocompromised by malignancies or chemotherapy, CMV involving the ovaries with HIV infection has not been noted heretofore. The patient described here had intractable pelvic and rectal pain, which can be explained, in part, by the extensive CMV oophoritis. The findings of CMV in the uterus and fallopian tube, while interesting, were not associated with the extensive inflammation seen in the ovaries.

With the increasing number of female AIDS patients, we may postulate that CMV oophoritis, as well as CMV infection of other areas of the genital tract, will be seen more often. However, in a 5-year period, a review of 12 autopsies in the Department of Pathology, University of Rochester, in female AIDS patients, 4 of whom had disseminated CMV, no such involvement of the ovaries or other genital tract structures was seen. In view of the difficulty in diagnosing this disorder, CMV oophoritis secondary to CMV infection should be included in the differential diagnosis of unexplained pelvic pain in female AIDS patients.

### ACKNOWLEDGMENTS

The authors are indebted to George Abbott, M.D., Department of Pathology, Highland Hospital, Rochester, NY, for his expert preparation of the microscopic sections and photographs.

### REFERENCES

1. Drew W. Cytomegalovirus infection in the patients with AIDS. Clin Infect Dis 14:608–614, 1992.
2. Armstrong D, Gold J, Drysanski J, et al. Treatment of infections in patients with acquired immunodeficiency syndrome. Ann Intern Med 103:738–743, 1985.
3. Lang D. Cytomegalovirus infection. In Wyngaarden J and Smith L (eds): Cecil: Textbook of Medicine, 18th ed. Philadelphia: WB Saunders, 1988, pp 1784–1786.
4. Iwasaki T, Sakuma T, Takano N, et al. Cytomegalovirus oophoritis with cortical necrosis during remission of acute lymphocytic leukemia. Acta Pathol Jpn 38:1069–1076, 1988.
5. Tashiro J, Goto J, Nasu M. A clinicopathological study on cytomegalovirus infection. J Jpn Assoc Infect Dis 63:1171–1177, 1989.
6. Meyers J, Flournoy N, Thomas E. Nonbacterial pneumonia after allogeneic marrow transplantation: A review of ten years' experience. Rev Infect Dis 4:1119–1132, 1982.
7. Schooley R, Hirsch M, Colvin R, et al. Association of herpesvirus infections with T-lymphocyte-subset alterations, glomerulopathy, and opportunistic infections after renal transplantation. N Engl J Med 308:307–313, 1983.
